# Rosiglitazone modulates collagen deposition and metabolism in atherosclerotic plaques of fat-fed ApoE-knockout mice

**DOI:** 10.3892/etm.2015.2711

**Published:** 2015-08-25

**Authors:** MINGXUE ZHOU, HAO XU, WEIHONG LIU, HONGXU LIU

**Affiliations:** 1 Beijing Institute of Traditional Chinese Medicine, Beijing Hospital of Traditional Chinese Medicine Affiliated to Capital Medical University, Beijing 100010, P.R. China; 2 Department of Cardiovascular Medicine, Xiyuan Hospital, Academy of Chinese Medical Sciences, Beijing 100091, P.R. China; 3 Department of Cardiovascular Medicine, Beijing Hospital of Traditional Chinese Medicine Affiliated to Capital Medical University, Beijing 100010, P.R. China

**Keywords:** rosiglitazone, plaque stability, atherosclerosis, collagen deposition and metabolism

## Abstract

Abnormal collagen deposition, as well as collagen metabolism, plays a crucial role in the formation and progression of vulnerable atherosclerotic plaques (VAPs), which are susceptible to rupture. According to our previous findings, rosiglitazone, a thiazolidinedione, can promote the stability of atherosclerotic plaques in fat-fed ApoE-knockout mice; however, it is unknown whether it can modulate collagen deposition and metabolism in VAPs. The present study was designed to determine the effect of rosiglitazone on collagen deposition and metabolism in the plaques of fat-fed ApoE-knockout mice. Following 13 weeks of the high-fat diet, the mice were randomized into three groups (10 mice/group) and intragastrically administered rosiglitazone, simvastatin and distilled water, respectively, for a further 13 weeks. The category of the collagen present in the plaques was evaluated using the picro-Sirius red polarization method. Additionally, the protein expression of matrix metalloproteinase 9 (MMP-9) and tissue inhibitor of metalloproteinase-1 (TIMP-1) in the plaques was determined using immunohistochemistry. The results showed that rosiglitazone reduced the lipid to collagen and type III to type I collagen ratios in the plaques, and these reductions were correlated with the reduction in the plaque MMP-9 to TIMP-1 ratio. These results suggest that rosiglitazone can modulate collagen deposition and metabolism and promote the stabilization of VAPs.

## Introduction

Arterial diseases associated with atherosclerosis are the leading cause of morbidity and mortality worldwide. Despite the development of anti-inflammatory treatments and agents to lower lipid levels, the use of which can reduce the incidence of acute coronary syndromes (ACSs), preventive strategies that specifically target the mechanisms causing plaque destabilization remain elusive (1). It is widely known that type 2 diabetes is considered a ‘risk equivalent’ for cardiovascular disease. Rosiglitazone, a thiazolidinedione, has been investigated as a potential therapeutic agent for the prevention of cardiovascular disease, such as atherosclerosis (2–4).

Collagen is the main component of the extracellular matrix (ECM) in atherosclerotic plaques. It is not only a simple supporting structure but also exerts numerous bioactivities, such as storing lipids, secreting cellular factors and promoting smooth muscle cell proliferation (5–7), and is associated with the progression of atherosclerosis (8). Additionally, collagen can be found as the main component of fibrous caps, providing tensile strength. The loss of collagen can result in structural weakness and reduces the resistance to the mechanical stresses associated with systole (9). The consequence of this structural weakness is plaque rupture, which is the key event in the initiation of coronary thrombosis and, therefore, ACSs, such as unstable angina and myocardial infarction (10). In vulnerable atherosclerotic plaques (VAPs), types I and III collagen are the most evident collagen categories, and the former is the most important collagen to endure the loading in the plaque fibrous cap.

In human atherosclerosis, it is believed that the increased activity of matrix metalloproteinase 9 (MMP-9) can lead to the upregulation of collagen deposition, possibly through transforming growth factor-β activation (11). In addition, MMP-9 can degrade collagen fractions in atherosclerotic plaques to promote the formation and rupture of the plaques. MMP-9 is specifically inhibited by tissue inhibitor of metalloproteinase-1 (TIMP-1) outside the cell. When the increasing expression of MMP-9 surpasses that of TIMP-1, the rate of collagen degradation will surpass that of collagen synthesis; therefore, the MMP-9/TIMP-1 ratio can be used to evaluate the stability of atherosclerotic plaques (12,13).

Our previous results showed that rosiglitazone could promote the stability of atherosclerotic plaques in fat-fed ApoE-knockout mice by modifying the plaque composition, as well as by decreasing the number of buried fibrous caps (14); however, the effect of rosiglitazone on collagen deposition and metabolism in the plaque is unknown. The aim of this study, therefore, was to determine the effect of rosiglitazone on collagen metabolism in the plaques of fat-fed ApoE-knockout mice.

## Materials and methods

### 

#### Animals

Male ApoE-knockout mice (n=30; age, 8 weeks; weight, 18–20 g) with a C57BL/6J background were introduced and bred by the Laboratory Animal Center of Peking University Health Science Center (Beijing, China). All animals were housed, cared for and used in procedures in accordance with the guidelines and regulations of the University of Bristol (Bristol, UK) and the United Kingdom Home Office.

#### Husbandry

The ApoE-knockout mice were sustained on a high-fat diet that contained 21% (wt/wt) fat from lard supplemented with 0.15% (wt/wt) cholesterol (Special Diet Services, Witham, UK) (15) for 26 weeks. All mice were inspected on a regular basis, with at least one inspection every 24 h.

#### Drug treatment

After the first 13 weeks of being fed the high-fat diet, the ApoE-knockout mice were randomly assigned to one of three groups (10 mice/group) and treated intragastrically with rosiglitazone (0.60 mg/kg per day), simvastatin (9.01 mg/kg per day), both purchased from GlaxoSmithKline Pharmaceutical Co., Ltd. (Tianjin, China), or distilled water (control group) for the remaining 13 weeks of the high-fat diet. The dose selection was based on the equivalent clinical doses in humans, using the conversion coefficient of 9.01; therefore the doses were calculated using the following formula: Dose in mice = clinical dose in human × 9.01. In clinical practice, rosiglitazone and simvastatin are administered to patients at doses of 0.067 and 1 mg/kg per day, respectively; applying these doses to the above formula gave mouse doses of 0.60 mg/kg per day for rosiglitazone and 9.01 mg/kg per day for simvastatin. The drugs were diluted using distilled water. The distilled water consumption of the mice was monitored twice weekly, and the drug concentration was adjusted when necessary.

#### Histology

At the end of the 13-week drug treatment period, the mouse hearts were removed and embedded in paraffin. Six serial 5-µm sections were cut at 50-µm intervals from the cross section of the cardiac base until the ascending aorta appeared. For the quantitative analysis of the atherosclerotic lesions, four 5-µm sections were selected and quantified using a previously described method (16). Up to six of the 5-µm sections per mouse were morphometrically and immunohistochemically analyzed. The collagen and foam cells in the plaques were stained using a modified Movat pentachrome stain. The area of lipid content within the atherosclerotic plaque was calculated using the following formula: Area of lipid content = area of extracellular lipid core + area of foam cells in the atherosclerotic plaque.

#### Determination of collagen category

The category of collagen was evaluated using the picro-Sirius red polarization method. Following staining, type I collagen appears red or yellow and type III collagen appears green under a BH-2 polarimicroscope (Olympus Corporation, Tokyo, Japan).

#### Immunohistochemistry

The serial 5-µm paraffin sections were dewaxed and rehydrated, and the endogenous peroxidase activity was terminated by incubation with 3% hydrogen peroxide. The sections were subsequently blocked using 20% (v/v) goat serum in phosphate-buffered saline, prior to being incubated overnight at 4°C with mouse MMP-9 monoclonal antibody (1:100; sc-21773; Santa Cruz Biotechnology, Inc., Santa Cruz, CA, USA) and rabbit TIMP-1 polyclonal antibody (1:200; 10753-1-AP; Proteintech Group, Inc., USA). The sections were then incubated with the polyclonal mouse/rabbit secondary antibodies (k5007; 1:200; Dako, Glostrup, Denmark). Areas that were found to be positive for the target protein expression were counted and expressed as a percentage of the whole plaque area. Negative controls were established by replacing the primary antibody with either mouse or rat IgG at the same dilution. The analysis of the positive sections was conducted in a blinded manner using Image-Pro Plus image analysis software (Media Cybernetics, Inc., Rockville, MD, USA).

#### Statistical analysis

Data are expressed as the mean ± standard deviation, and comparisons between the groups were conducted using one-way analysis of variance. For the analysis of the correlation between the lipid to collagen, type III to type I collagen and MMP-9 to TIMP-1 ratios in the rosiglitazone-treated group, the data were assessed using correlation analysis. In all cases, P<0.05 was considered to indicate a statistically significant difference.

## Results

### 

#### A high-fat diet induces the formation of VAPs

**Figure 1.**
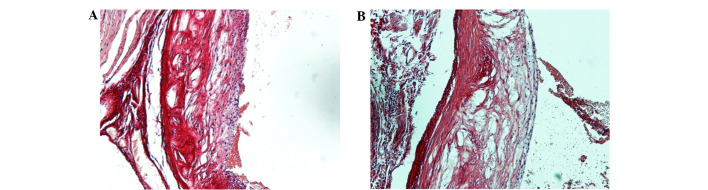
A high-fat diet induces the formation of vulnerable plaques. (A and B) Pathological morphology of the atherosclerotic plaque in the aortic root of ApoE-knockout mice (A) 13 weeks and (B) 26 weeks after high-fat feeding (stain, hematoxyling and eosin; magnification, x200).

Atherosclerotic plaques could be clearly observed in the aortic roots of the ApoE-knockout mice after 13 weeks of the high-fat diet (Fig. 1A). After a further 13 weeks of the high-fat diet, the plaques exhibited the typical morphological features of VAP, including a large lipid core and thin fibrous cap (Fig. 1B).

#### Effect of rosiglitazone on plaque stability

**Figure 2.**
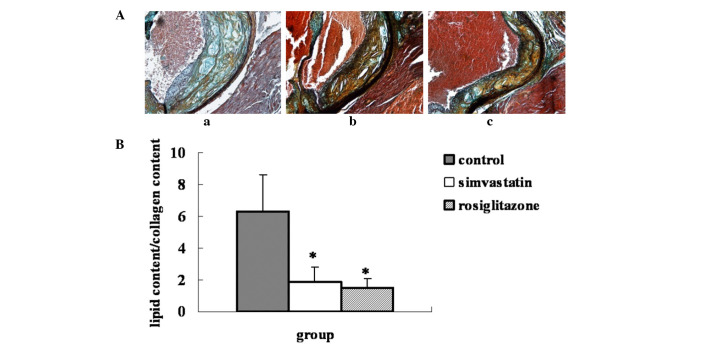
Effect of rosiglitazone treatment on plaque composition in the aortic root of ApoE-gene knockout mice. (A) Comparison among the three groups of the plaque content in the aortic root of ApoE-knockout mice treated with the indicated drug for 13 weeks: (a) Control, (b) simvastatin (9.01 mg/kg per day) and (c) rosiglitazone (0.6 mg/kg per day). Treatment with rosiglitazone for 13 weeks not only decreased the lipid core in the plaques but also increased the collagen content compared with the control group (stain, Movat; magnification, x200). (B) Comparison of the ratio of lipid to collagen content in the aortic plaques of ApoE-knockout mice among the groups following drug treatment for 13 weeks. Both rosiglitazone and simvastatin treatment for 13 weeks decreased the ratio of lipid to collagen content. Values are expressed as the mean ± standard deviation (n=10). *P﹤0.01 vs. control.

Compared with the plaques of the control group, the ratio of lipid to collagen content in the plaques of the rosiglitazone-treated group was significantly decreased by 75.8% (P=0.0004 vs. control), while in the simvastatin group the ratio was decreased by 70.6% (P=0.001 vs. control). No statistical difference in plaque stability was observed between the two drug-treated groups (Fig. 2).

#### Effect of rosiglitazone on the category of collagen in the plaque

**Figure 3.**
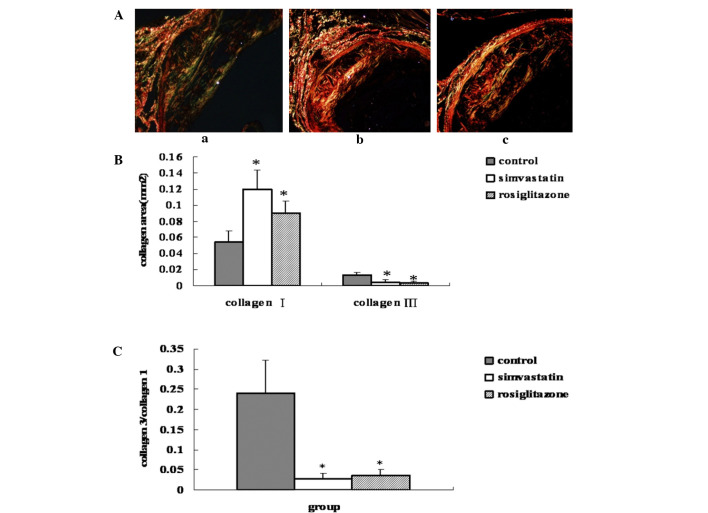
Effects of rosiglitazone treatment on the content of the collagen types in the aortic root of ApoE-gene knockout mice. (A) Comparison among the groups of collagen types within the plaque in the aortic root of ApoE-knockout mice following drug treatment for 13 weeks: (a) Control, (b) simvastatin (9.01 mg/kg per day) and (c) rosiglitazone (0.6 mg/kg per day). Red-stained regions represent type I collagen and yellow-stained regions represent type III collagen in the plaque (picro-Sirius red polarization staining; magnification, x200). (B) Treatment with rosiglitazone for 13 weeks increased the content of collagen type I and decreased the content of collagen type III in the plaques compared with the control group. (C) Comparison among the groups of the ratio of collagen type III to type I content in the aortic plaques of ApoE-knockout mice following drug treatment for 13 weeks. Both rosiglitazone and simvastatin treatment for 13 weeks decreased the ratio of collagen type III to type I content in the plaques. Values are expressed as the mean ± standard deviation (n=10). *P﹤0.01 vs. control.

As shown in Fig. 3, rosiglitazone treatment for 13 weeks increased the type I collagen content in the plaque by 66.8% (P=0.01 vs. control), while simvastatin treatment led to a 1.2-fold increase (P=0.016 vs. control) compared with the control group. In addition, treatment with rosiglitazone and simvastatin decreased the type III collagen content in the plaque by 76.6% (P=0.0005 vs. control) and by 69.4% (P=0.0006 vs. control), respectively. The ratio of type III to type I collagen in the rosiglitazone treated group was, therefore, significantly decreased by 85% (P=0.0007 vs. control), while in the simvastatin-treated group the ratio was decreased by 88.6% (P=0.01 vs. control). No statistically significant differences were observed between the two drug-treated groups (P>0.05) (Fig. 3).

#### Effect of rosiglitazone on enzymes modulating collagen metabolism

**Figure 4.**
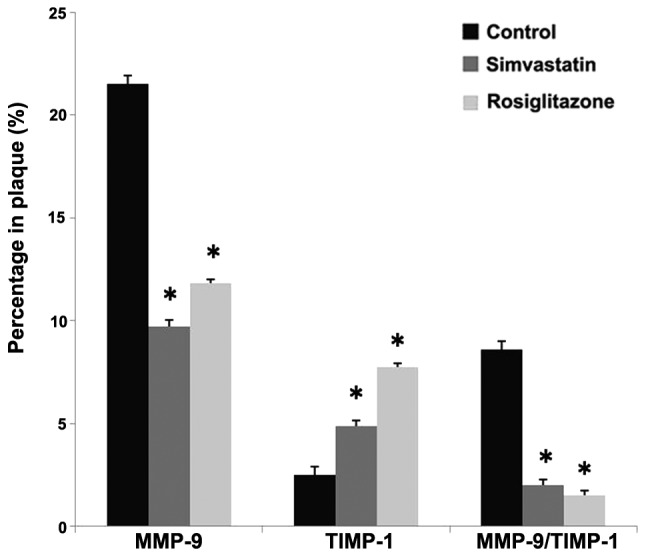
Effect of rosiglitazone treatment on the expression of MM9-1 and TIMP-1 in plaques in the aortic root of ApoE-knockout mice. Rosiglitazone treatment for 13 weeks decreased the positive expression of MMP-9 in the plaques by 45.5% (P=0.0003) and increased the positive expression of TIMP-1 by 2.1-fold (P=0.0003) compared with the control group. Rosiglitazone treatment also decreased the MMP-9/TIMP-1 ratio by 82.3% (P=0.0002). MMP-9, matrix metalloproteinase 9; TIMP-1, tissue inhibitor of metalloproteinase-1. *P﹤0.01 vs. control.

As in Fig. 4, rosiglitazone and simvastatin treatment decreased the expression of MMP-9 by 45.5% (P=0.0003 vs. control) and 54.7% (P=0.0005 vs. control), respectively. By comparison, after rosiglitazone treatment for 13 weeks the expression of TIMP-1 in the plaque was increased by 2.1-fold compared with the control group (P=0.0003 vs. control), while the TIMP-1 expression in the simvastatin group was increased by 94.5% (P=0.0004 vs. control). Rosiglitazone treatment therefore decreased the MMP-9 to TIMP-1 ratio by 82.3% (P=0.0002 vs. control) and simvastatin treatment decreased the ratio by 76.7% (P=0.0003 vs. control) compared with the control group. No statistically significant differences were observed between the two drug-treated groups (P>0.05).

#### Correlation analysis for ratios of enzyme expression, collagen category and plaque content in the rosiglitazone group

The MMP-9 to TIMP-1 ratio in the rosiglitazone-treated group was found to be significantly correlated with the ratio of lipid to collagen content in the plaque (γ=0.957, P<0.01). In addition, the ratio of MMP-9 to TIMP-1 in the rosiglitazone-treated group was significantly correlated with the ratio of type III to type I collagen in the atherosclerotic plaque (γ=0.863, P<0.01).

## Discussion

As a member of the thiazolidinedione class of drugs, rosiglitazone can reduce the plasma levels of C-reactive protein and soluble cluster of differentiation 40 ligand (17,18), and pre- or post-treatment with rosiglitazone has been shown to reduce aortic expansion and rupture in an angiotensin II-induced hypercholesterolemic mouse model (19). Furthermore, the reduction in the rupture of lesions in the mice pretreated with rosiglitazone was coincident with a downregulation in the expression of inflammatory mediators (19). Our previous study showed that rosiglitazone could promote the stability of atherosclerotic plaques in fat-fed ApoE-knockout mice by reducing the vulnerability index, as well as the average quantity of buried fibrous caps, which may have been associated with its anti-inflammatory effects (14); however, the effect of rosiglitazone on collagen deposition and metabolism in atherosclerotic plaque was unclear. The present study indicated that rosiglitazone could modulate collagen deposition and metabolism in the atherosclerotic plaques of fat-fed ApoE-knockout mice, which may represent an important mechanism underlying the rosiglitazone-induced stability of atherosclerotic plaques.

Plaque stability has been suggested to be dependent upon the plaque composition and the state of the fibrous cap (20). Collagen is the main component of the ECM and plays a crucial role in keeping the atherosclerotic plaque intact and stable. Collagen is responsible for the majority of the tensile strength of the intima, which is significant as the rupture of the fibrous cap is believed to be the event that immediately precedes atherosclerosis-related arterial thrombosis. The ability of the atherosclerotic plaque to resist mechanical tensile strength is reduced by an increase in the lipid component, particularly cholesterol ester, of the plaque; however, an increase in the collagen component of the plaque, particularly in the fibrous cap, can keep the fibrous cap intact and promote the resistance of mechanical tensile strength. The ratio of lipid to collagen content is therefore an important index for the evaluation of plaque stability (21). In the present study, the results showed that rosiglitazone could reduce the ratio of lipid to collagen, as well as the lipid core area, in the atherosclerotic plaque.

In VAPs, the category of collagen deposited in the plaque is another important factor. Types I and III collagen are the main collagen categories. Type I collagen, a type of mature collagen, is the important collagen for enduring the loading on the plaque fibrous cap due to its enhanced ability to resist mechanical strength; type I collagen gene expression is focal and particularly prevalent in the fibrous cap (22). By comparison, type III collagen, a type of immature collagen, can reduce the stability of atherosclerotic plaques due to its poor resilience. The ratio of type III to type I collagen in an atherosclerotic plaque may therefore, to some degree, reflect the pathological atherosclerotic changes. The present results indicated that rosiglitazone, in a clinically relevant dose, could modulate the collagen deposition in atherosclerotic plaques by reducing the ratio of type III to type I collagen, which is favorable for promoting plaque stability.

In the fibrous cap of an atherosclerotic lesion, collagen represents the ECM component that is responsible for maintaining the structural integrity; therefore, the balance between the synthesis and degradation of this collagen appears to be a critical factor in plaque stability. Excessive collagen deposition can result in stenosis of the vasculature, whereas too little collagen in an atherosclerotic plaque can render the plaque vulnerable; therefore, maintaining the appropriate levels of collagen deposition and metabolism in atherosclerotic plaques is of particular importance.

MMPs form a large family of enzymes, which can collectively degrade all components of the ECM. Types I and III collagen are degraded by MMPs, the activity of which plays an important role in the inflammatory reaction. There is an increased rate of MMP formation in ruptured atherosclerotic plaques (23). MMP-9 is an important MMP secreted by inflammatory cells, such as macrophages. Although it cannot directly degrade types III and I collagen, it can thoroughly decompose the collagen fragments of the fibrous cap in order to promote the formation and rupture of the VAP. Previous studies have suggested that MMP-9 is the key factor in the induction of plaque rupture, and its expression strongly correlates with lesional instability and the clinical manifestations of atherosclerosis (24–26). The effect of MMP-9 overexpression on plaque rupture was originally selected as a focus of study due to the ability of MMP-9 to degrade elastin and cleaved collagen, both of which abound in the ECM of fibrous caps of advanced atherosclerotic lesions.

MMP activity is, at least partially, controlled by a family of endogenous inhibitors known as TIMPs. TIMP-1 is believed to be the key member in the TIMP family with regard to the regulation of MMP-9 activity, and MMP-9 is secreted as a complex with TIMP-1 when expressed by macrophages (13). The MMP-9 to TIMP-1 ratio also affects the reactivity of MMPs, to a certain extent, so it reflects the balance or imbalance between the degradation and synthesis of collagen. MMP-9 to TIMP-1 ratios can therefore be used to evaluate the stability of atherosclerotic plaques (12,13). The present results showed that rosiglitazone could reduce the MMP-9 to TIMP-1 ratio in atherosclerotic plaques and that the reduction was significantly correlated with the reduction in the ratios of lipid to collagen content and type III to type I collagen in the plaques. We therefore concluded that rosiglitazone could modulate collagen deposition to promote the stability of the atherosclerotic plaque by reducing the MMP-9 to TIMP-1 ratio.

In conclusion, rosiglitazone, as an insulin sensitizer, can stabilize atherosclerotic plaques by modulating collagen deposition and metabolism in the plaques of fat-fed ApoE-knockout mice. Further studies are required to elucidate the precise mechanism.
